# A Study on Knowledge, Awareness, and Medication Adherence in Patients with Hypertension from a Tertiary Care Centre from Northern Sri Lanka

**DOI:** 10.1155/2017/9656450

**Published:** 2017-11-02

**Authors:** S. Pirasath, T. Kumanan, M. Guruparan

**Affiliations:** ^1^Professorial Medical Unit, Faculty of Medicine, University of Jaffna, Jaffna, Sri Lanka; ^2^Teaching Hospital Jaffna, Jaffna, Sri Lanka

## Abstract

**Objective:**

To assess the patient's knowledge and awareness about hypertension and adherence to antihypertensive medication among hypertensive patients with validated Morisky questionnaires in a tertiary care centre of northern Sri Lanka.

**Methods:**

A cross-sectional descriptive comparative study was carried out at Teaching Hospital Jaffna, from January 2017 to April 2017. Hypertensive patients were recruited by systematic randomized controlled sampling and interviewed with validated Morisky questionnaires to assess their knowledge about hypertension. Data were analyzed using SPSS (version 21) analytical package.

**Results:**

73 of 303 patients were males. 69.9% of patients had adequate knowledge about hypertension. 40.5% of patients were unaware of their disease status. 75.8% of patients could not recall their blood pressure values at the time of diagnosis. 72.3% of patients were unaware of their values of blood pressure during their last outpatient clinic visit. 48.2% of patients had awareness of target organ damage due to hypertension (kidney, 72, 23.7%; heart, 128, 42.2%; brain, 140, 46.7%; eye, 42, 13.8%). Most of the patients had poor drug compliance. The most common reasons for nonadherence were forgetfulness (70, 23.1%) and interruptions of daily routine (53, 17.5%).

**Conclusion:**

The knowledge about hypertension among majority of patients was reasonable. But they were unaware of their disease status. The drug compliance among them was poor. Forgetfulness and interruptions of daily routine were common reasons attributed for nonadherence.

## 1. Introduction

Hypertension is a major public health burden and is part of an epidemiological transition from communicable to noncommunicable diseases globally [1]. It is an important risk factor for stroke, coronary heart diseases, peripheral vascular disease, heart failure, and chronic kidney disease [1]. The aging, urbanization, sedentary lifestyle, obesity, ethanol consumption, and excess salt intake are the contributing factors for epidemiological transition of hypertension in world [2]. A cost-effective use of health services such as increasing the knowledge and awareness, detection, treatment, and control of hypertension (HT) is needed among public in developing countries, particularly about the risks associated with uncontrolled blood pressure [3]. Screening for elevated systolic blood pressure (SBP) has been identified as an important medical challenge in the prevention and treatment of hypertension [4]. This study was aimed at assessing the patient's knowledge and awareness about hypertension and adherence to antihypertensive medication among hypertensive patients in a tertiary care centre of northern Sri Lanka.

## 2. Methods

### 2.1. Study Design

This was a cross-sectional descriptive comparative study.

### 2.2. Study Area

 The study area used was medical outpatient clinics of Teaching Hospital Jaffna of northern region of Sri Lanka.

### 2.3. Study Settings

The hypertensive patients attending the general medical clinics, Teaching Hospital Jaffna, were included in this study. The Teaching Hospital Jaffna is the only tertiary care centre in northern Sri Lanka. Majority of the hypertensive patients from all over the Jaffna Peninsula attended the general medical clinic of Teaching Hospital Jaffna. Hence we hope that the knowledge, awareness, adherence, and lifestyle practices of the community will be represented in the study.

### 2.4. Sample Size

303 patients were recruited from the all four medical outpatient general medical clinics of Teaching Hospital in Jaffna.

### 2.5. Sampling Method

The eligible respondents were selected by systematic randomized controlled sampling method.

### 2.6. Study Period

The study was conducted for a period of 4 months from January 2017 to April 2017.

### 2.7. Ethics

The ethical clearance was obtained from Ethical Review Committee, Faculty of Medicine, University of Jaffna, Sri Lanka. After proper approval, three hundred and three patients with their informed written consent were included in the study. The subjects were interviewed to gather clinical and demographic details.

### 2.8. Diagnosis of Hypertension

Hypertension was diagnosed with values of SBP/DBP > 140/90 mmHg, measured by a standard mercury sphygmomanometer in at least two occasions using standardized methods.

### 2.9. Inclusion and Exclusion Criteria

Those who were eligible for inclusion into this descriptive cross-sectional, qualitative phenomenological survey were a cohort of 303 adult male and female hypertensive patients drawn from four general medical clinics, Teaching Hospital Jaffna. Participants with a systolic blood pressure (SBP) above 140 mmHg and/or diastolic blood pressure (DBP) above 90 mmHg who were willing to participate in the study were included, whereas patients who were pregnant, below 18 years of age, and mentally incompetent were excluded.

### 2.10. Development of Questionnaires

#### 2.10.1. Hypertension Fact Questionnaire

Hypertension Fact Questionnaire was designed as a tool, using the existing literature, practicing physicians, and cardiologists to assess the knowledge and awareness among the hypertensive patients. The questionnaire was initially designed in English and then translated to Tamil language. The questionnaire consists of 14 and 9 questions to assess the patients' knowledge and awareness on hypertension, respectively. The patients who met the inclusion criteria were interviewed to assess their knowledge and awareness about hypertension.

#### 2.10.2. Morisky Medication Adherence Scale

Their medication adherence behavior and the reasons for nonadherence were studied using Morisky self-report measure of medication adherence. Morisky scale consists of 8-point questions (never/rarely/sometimes/often/always) and a set of open-ended questions regarding reasons for nonadherence. Scores for the scale range within low adherence (<6), medium adherence (6 to <8), and high adherence (=8). The higher scores are indicative of worse adherence. All subjects who answered “yes” for at least one question were considered as nonadherent.

### 2.11. Data Analysis

Data were entered in Microsoft Excel sheet and were analyzed using SPSS (version 21) analytical package. Baseline results were presented as counts and percentages and as mean ± SD for continuous variables. A *P* < 0.05 will be considered significant.

## 3. Results

The basic demographic and pattern of risk factors associated with hypertension were shown in Table 1.

### 3.1. Knowledge and Awareness of Hypertension among Patients

The knowledge and awareness of hypertension were tested among 303 hypertensive patients with validated questionnaires and results were shown in Tables 2 and 3, respectively. 69.9% of patients had adequate knowledge about hypertension. However, 30.1% of patients had minimum knowledge about hypertension. Even though they had good knowledge about hypertension, 40.5% of patients were unaware of their disease status. 75.8% of patients had not known their values of blood pressure at time of diagnosis. 72.3% of patients were unaware of their values of blood pressure at the time of their last visit. 75% of patients who knew the values of blood pressure at time of last visit thought wrongly that their blood pressure control was adequate.

48.2% of patients had awareness of target organ damage due to hypertension (Kidney, 72, 23.7%; heart, 128, 42.2%; brain, 140, 46.7%; eye, 42, 13.8%). 80% of patients were concerned that high blood pressure is a serious health issue. Almost all patients (99%) thought that taking medicine is key to control the blood pressure.

### 3.2. Adherence and Reasons for Nonadherence

All patients were interviewed about their drug adherence and possible reasons for nonadherence. The questionnaire contains eight questions, of which seven are yes or no type questions and the eighth one is a multiple-choice question. The drug adherence of the patients was shown in Table 4. These patients were asked to understand their reason for nonadherence to the treatment for hypertension. There were 15 universal reasons that were included in the questionnaire and responses were shown in Table 5. Almost all patients (99%) thought that taking medicine plays a key role in controlling the blood pressure. But most patients (84.5%) had poor drug compliance. The most common reasons for nonadherence were forgetfulness (70, 23.1%) and interruptions of daily routine (53, 17.5%).

## 4. Discussion

Hypertension remains a challenging medical condition among the noncommunicable diseases of ever growing population. Efforts to control HT include increasing public knowledge and awareness about the risks associated with high BP. We conducted this cross-sectional descriptive survey to evaluate the current status of hypertension knowledge, awareness, and adherence in a group of hypertensive patients from a distinct community.

The National High Blood Pressure Education Program was launched to improve the public's knowledge of HT in 1972 [5]. Data from the National Health and Nutrition Examination Survey (NHANES II and NHANES III) reported an increase in BP awareness during the time period 1976–1991 from 51% to 73% [6]. Some other studies have assessed HTN knowledge and awareness in the general population [7] and hypertensive population [8] showing a decreased level of knowledge and awareness. In our study most patients (69.9%) had adequate knowledge about hypertension. Almost all patients were aware of their HT with 86.1% reporting that a doctor or healthcare provider had told them that they have HT. But most patients (40.5%) were unaware of current status of their disease. These findings are consistent with NHANES III data suggesting that there has been an increase in BP awareness [5].

Recent reports have suggested that hypertension knowledge is related to BP control [9]. SBP is a strong independent risk factor for cardiovascular disease. It is important to assess the extent to which patients are aware of the importance of controlling their SBP levels. 75.8% of patients had not known their values of blood pressure at time of diagnosis. 72.3% of patients were unaware of their values of blood pressure at time of last visit. 75% of patients who knew the values of blood pressure at time of last visit were in a false assumption that their blood pressure control was satisfactory. Patients were unaware that SBP is important in BP control and reported that physicians did not emphasize the significance of high SBP levels. These findings suggest the need for education of patients by physicians and other primary healthcare providers related to the importance of elevated SBP and cardiovascular risk. Many patients could not recall their BP level at time of diagnosis (75.8%) and at last clinic visit (72.3%). Even though the patients knew their BP level at time of diagnosis (24.2%) and at last clinic visit (27.7%), they were not in a position to accurately classify their blood pressure level. These findings suggest that patients' perception of their BP level does not reflect their actual readings except for the majority of those with controlled BP. Further, 54.4% of patients reported that their values were in the normal range, but in fact the values were elevated. Control of SBP and improved drug compliance have been achieved through an education program that could emphasize at least to a certain extent the fact of the concept “knowing high BP.” This recent research clearly illustrates the need to improve hypertension knowledge and awareness in order to improve the medication adherence and optimum blood pressure control.

In terms of Farquhar's model of behavioral change [10], our findings suggest that most patients had sufficient knowledge about HT, but only a few showed real motivations (wish and attempt) to change. We observed that few patients have reached the stages of skills and action whereby individuals actively engage in a new behavior during conservation with them. An opportunity exists to use patient-reported sources for HT information in order to disseminate HT information. In this study, physicians, other healthcare providers, mass media, print, and video materials were identified as important sources of information to improve hypertension knowledge and awareness as reported by the patients.

Hypertension presents a major area of intervention because it is amenable to control through both nonpharmacological lifestyle modification and medications. Pharmacological treatment for hypertension has been shown to be effective in decreasing BP and subsequently cardiovascular events [11]. Lifestyle measures for lowering BP include cessation of smoking, moderation of or abstaining from consumption of ethanol, restricting the intake of salt, regular physical exercise, healthy eating pattern, and losing weight [12]. Lifestyle interventions have pivotal role in reducing the number of medications among hypertensive patients and preventing the risk of developing hypertension among normal population. Several models have been proposed to account for health behaviors and sustained behavioral changes [13]. Although they differ in their content and perspective, models for behavior change emphasize the importance of evaluating the perceptions, attitudes, beliefs, and outcome expectations of individuals as crucial elements to understand observed behaviors and to guide behavioral change [14].

Poor compliance is a common and important challenge leading to treatment failure in clinical practice [15]. It is a barrier to effective management of hypertension. Most of patients (84.5%) had poor compliance of drugs in our study. The forgetfulness and interruptions of daily routine were common reasons for nonadherence. This study was aimed at assessing the patient's knowledge and awareness about hypertension and adherence to antihypertensive medication among hypertensive patients in a tertiary care centre from northern Sri Lanka. These data emphasize the need to maximize the efficiency of hypertension prevention and control programs so that delay in achieving optimum blood pressure control is minimized in countries experiencing recent emergence of hypertension as a major public health problem. The results of this study in selected patients attending general medical clinics of a tertiary care centre of a region may not reflect the reality of the general population of Sri Lanka.

## 5. Conclusion

The patients had adequate knowledge about hypertension. But they were unaware of their disease status. Most patients had poor drug compliance. The forgetfulness and interruptions of daily routine were the most common reasons for nonadherence among the patients.

## Figures and Tables

**Table 1:** Selected demographic and risk factors among hypertension.

Selected variables	Numbers	Percentage
Gender		
Male	73	24.1
Female	230	75.9

Age (years)		
30–39	10	03.3
40–49	66	21.8
50–59	85	28.1
60–70	105	34.7
>70	37	12.1

Body mass index, kg/m2		
Normal	66	21.8
Overweight	194	64.0
Obese	43	14.2

Alcohol consumption (mL/d)		
Nondrinkers (0 or occasional)	238	78.5
Moderate drinkers (1–100)	45	14.9
Heavy drinkers (>100)	20	06.6

Reported level of exercise, score		
Low	265	87.5
Intermediate	25	08.2
High	13	04.3

Smoking (cigarettes/d)		
Nonsmokers (0)	242	79.9
Smokers (>1)	61	20.1

Serum total cholesterol (mmol/L)		
Normal (5.2)	73	24.1
Borderline (5.2–6.5)	194	64.0
High (>6.5)	36	11.9

**Table 2:** Patient's knowledge of hypertension.

Questions	Yes	No	Yes (%)	No (%)
Knowing normal values of BP as 120/80 mmHg	220	83	72.6	27.4
Increase in BP > 140/90 mmHg called HT	219	84	72.3	27.7
HT can progress along with the age	232	71	76.4	23.4
Both sexes have equal chance of developing HT	161	142	53.1	46.9
HT is a treatable condition	208	95	68.6	31.4
Risk of developing HT if there is a family history of HT	155	148	51.2	48.8
Aging is greater risk of HT	232	71	76.6	23.4
Smoking is a risk factor for HT	219	84	72.3	27.7
Eating fatty foods is a risk factor for HT	249	54	82.3	17.8
Overweight is a risk factor for HT	230	73	75.9	24.1
Regular physical exercise reduces HT	193	110	63.7	36.3
More salt consumption increases BP	225	78	74.3	25.7
Medication is alone in controlling HT	187	116	61.7	38.3
HT can lead to life-threatening condition	209	94	69.0	31.0

**Table 3:** Patient's awareness of hypertension.

Questions	Yes	No	Yes (%)	No (%)
Knowing about having hypertension	261	42	86.1	13.9
Knowing blood pressure values in diagnosing as hypertension	75	228	24.8	75.8
Knowing the values of target personal blood pressure	175	128	57.8	42.2
Controlling of blood pressure reduces your complications	202	101	66.7	33.3
Uncontrolled hypertension can lead to your organ's damage	146	157	48.2	51.8
Knowing values of blood pressure at recent visit	84	219	27.7	72.3
Thinking that HT is curable condition	284	19	93.7	6.3
Changing your lifestyle helps to lower your blood pressure	208	95	68.6	31.4
Improvement of your blood pressure over the last 12 months	228	75	75.2	24.8

**Table 4:** Patient's drug adherence of hypertension.

Questions	Yes	No	Yes (%)	No (%)
Do you sometimes forget to take your medication?	137	166	45.2	54.8

People sometimes miss taking their medication for reasons other than forgetting. Thinking over the past two weeks, were there any days when you did not take your medication?	86	217	28.4	76.6

Have you ever stopped or taken again medication without telling doctor?	43	260	14.2	85.8

When you leave/travel home, do you sometimes forget to take your medication?	83	220	27.4	72.6

Did you take your medicine yesterday?	246	57	81.2	18.8

When you feel like your health is under control, do you sometimes stop your medication?	47	256	15.5	84.5

Taking tablets every day is really unconvincing for some people. Do you ever feel hassled about sticking to your treatment plan?	46	257	15.2	84.8

How often do you have difficulty remembering to take all your medicine?				
Never/rarely—4	158	52.1		
Once a while—3	126	41.6		
Sometimes—2	19	6.3		
Never—1				

**Table 5:** Reasons for nonadherence of drugs.

Reasons	Numbers	(%)
Poor knowledge of disease and ignorance of long term treatment	27	8.9
Religious beliefs and cultural practices	31	10.2
Adverse drug reactions	26	8.6
Patient not believing that health depends on medicine	23	7.6
Worrying about taking medicine	33	10.9
Forgetfulness	70	23.1
Drug out of supply	24	7.9
Poor communication /insufficient patient information	35	11.6
Expenses (doctors' fees, transport, medicine, and hospitalization)	45	14.9
Interruptions of daily routine	53	17.5
Lack of reminders	35	11.6
Being busy or late for work	36	11.5
Being away on weekend/vacation	23	7.6
Too many medications to take	33	10.5
